# The Cost of Postponement of Bt Rice Commercialization in China

**DOI:** 10.3389/fpls.2019.01226

**Published:** 2019-10-08

**Authors:** Yan Jin, Dus̆an Drabik, Nico Heerink, Justus Wesseler

**Affiliations:** Wageningen University & Research, Wageningen, Netherlands

**Keywords:** Bt rice, cost of postponement, China, technology, trade

## Abstract

To maintain self-sufficiency in rice production and national food security, the Chinese government strongly supports research that aims at increasing the productivity of rice cultivation. Rice with genetic material from Bacillus thuringiensis (Bt rice) is transgenic rice that can reduce lepidopteran pest damage and the use of insecticides. It was developed in the 1990s and earned biosafety certificates in 2009. However, because of political reasons, its commercialization in China has been postponed, and, to date, Bt rice is not grown in China. We assess the opportunity cost of postponement of Bt rice commercialization in China between the years 2009 and 2019 and consider the external costs of pesticide use and potential technology spill-overs of Bt rice. We estimate the cost of postponement of Bt rice over the analyzed period to be 12 billion United States (US) dollars per year.

## Introduction

With only 6% of the world’s fresh water and 7% of its arable land, China has to nurture nearly a fifth of the world’s population (Wong and Chan, 2016). The arable land per capita in China decreased from 0.11 ha in 1990 to 0.09 ha in 2016, well below the world average of 0.19 ha per capita (World Bank, 2017b). Although rice is the predominant staple food in the country, the land allocated to its production decreased from 33.1 million ha in 1990 to 30.7 million ha in 2017 [National Bureau of Statistics of China (NBSC), 2018]. On the other hand, the amount of imported rice increased from 0.6 million metric tons in 2011 to 4.0 million tons in 2017, making China the biggest rice importer in the world (NBSC, 2018).

The United States (US) Census Bureau estimates that the Chinese population will reach 1.4 billion around 2026, which will further reduce the arable land per capita and increase the demand for rice. To maintain self-sufficiency in rice production and national food security, the Chinese government strongly supports research that aims to increase the productivity of rice cultivation. One of the priorities has, therefore, been the development of insect-resistant rice, such as rice with genetic material from *Bacillus thuringiensis* (Bt rice).

Bt rice is transgenic rice in which genes from the soil bacterium *Bacillus thuringiensis* have been transferred into the rice genome to reduce lepidopteran pest damage and the necessity of using insecticides (Huang et al., 2005). The yield of Bt rice can be up to 60% higher than conventional rice when no pesticides are used (Wang et al., 2010).

Chinese rice farmers apply more pesticides than farmers in most other countries (Huang et al., 2000). Huang et al. (2005) show, however, that Bt rice requires 80% less pesticide than conventional rice and reduces labor input (Rozelle et al., 2005). The simultaneous increase in production and reduction of input both contribute to the absolute increase of the total factor productivity of Bt rice, which is about 15% higher than conventional rice (Rozelle et al., 2005).

The adoption of Bt rice can also improve farmers’ health due to lower exposure to pesticides (Huang et al., 2015). Bt rice is also compatible with biological control and soil health management, although it should be noted that, to the best of the authors’ knowledge, no study examines its environmental effects at a larger scale or for a longer period (Cohen et al., 2008).

The cultivation of Bt rice in China requires special approval (Jin et al., 2019). The biosafety regulation system in China consists of three phases: field trials, environmental release trials, and preproduction trials. Before applying for field trials, Chinese scientists had spent 20 years investigating the thermal stability, digestibility, toxicity, and nutrient composition of Bt rice as well as the allergenicity of the Cry proteins it produces (Li et al., 2015). During various phases of the biosafety procedures, no food safety concern was raised. Bt rice is also found to be safe for aquatic ecosystems (e.g., Li et al., 2014) and has not shown any detrimental effects on non-target insect pests (Niu et al., 2017). It is expected to pose negligible risks to the non-target functional guilds in future large-scale Bt rice agroecosystems in China (Dang et al., 2017).

On October 22, 2009, China’s Ministry of Agriculture (MoA)^1^ issued biosafety certificates for two Bt rice lines (Cry1Ab/Ac Huahui No. 1 and Cry1Ab/Ac Bt Shanyou 63) (Chen et al., 2011). The issuance of the certificates indicates that the two lines are considered as safe as conventional rice, both to humans and the environment, and thus to be ready for commercialization. However, their official commercialization has been continuously postponed and is still pending. The biosafety certificates expired in 2014 but were renewed until the end of 2019.

The postponement of Bt rice commercialization is largely due to low public acceptance, like other genetically modified (GM) crops (e.g., Chen et al., 2014). Most Chinese business managers oppose food derived from GM crops because they fear lower profits (Deng et al., 2017). Although almost half of consumers know little about GM food, they believe it has adverse effects on human health and the environment (Qu et al., 2011). In addition, Chinese scientists do not show higher acceptance of GM food than non-scientists (Huang et al., 2017). Therefore, the government is hesitant to let China step forward as the first country to commercialize Bt rice.

More recently, however, the Chinese government has taken actions in policy support of the GM rice. In 2016, the “13th Five-Year Plan for Science and Technology Innovation” set an aim to push forward the commercialization of new domestic types of GM crops by 2020 (MoA, 2016).^2^ In the same year, the MoA revealed a roadmap for commercialization of transgenic crops, starting with cash crops “not for food use” (e.g., cotton) followed by crops for feed and industrial use (e.g., maize and soybeans), then non-staple food crops (e.g., sugar beets), and finally staple food crops (e.g., rice) [MoA, 2016; US Department of Agriculture (USDA), 2016].

Xie et al., 2017 estimate that each 1-year postponement of commercializing insect-resistant GM maize in China leads to the opportunity costs in the range of 4–14 billion US dollars for the overall economy. Moreover, postponement of commercializing Bt rice has high opportunity costs because of its foregone potential economic and environmental benefits. In this respect, it is important to consider the foregone benefits of lower pesticide use associated with Bt rice as well as its technology spill-overs on the international rice price. These effects have been neglected so far in the relevant literature, and no economic analysis of the cost of postponement (CoP) of Bt rice commercialization in China is available. Our paper aims to bridge this gap in the literature.

To achieve our objective, we combine the Economic Surplus Model (ESM) with the Pesticide Environmental Accounting (PEA) Tool. The ESM has been widely used to assess the benefits and costs of technical changes in agriculture (Alston et al., 1998). A sample of previous uses of the ESM includes Wesseler et al. (2017), who estimated the foregone benefits of delayed approval of staple crops (bananas, cow peas, and maize) in Africa; Bayer et al. (2010), who quantified the regulatory costs of Bt rice, Bt eggplants, ringspot-virus-resistant papayas, and virus-resistant tomatoes in the Philippines; and Krishna and Qaim (2007), who investigated the welfare and distributional effects of the introduction of the Bt technology among eggplant farmers and consumers in India.

We estimate the external costs of individual chemicals in rice production using the PEA, which is considered an appropriate tool for estimating the benefits of technologies replacing pesticides (Leach and Mumford, 2008; Prannetvatakul et al., 2013).

We provide essential information for different groups of stakeholders, including domestic and foreign policymakers determining the commercialization of GM crops in general, particularly Bt rice, and for businesses interested in investing in new biotechnology.

## Model for Assessment of the Policy

The ESM (Alston et al., 1998) is a tool for ex-ante assessment of the consequences of current technology improvements. We use it to calculate the welfare change between the counterfactual state of affairs had China commercialized Bt rice and the actual state of affairs due to the postponement of its commercialization. We model China as a large, open economy in rice trade. We set 2009 as the base year, since that is the year when Bt rice first received its biosafety certificate (MoA, 2009). Since then, Bt rice has been officially ready for commercialization.

**Figure 1:**
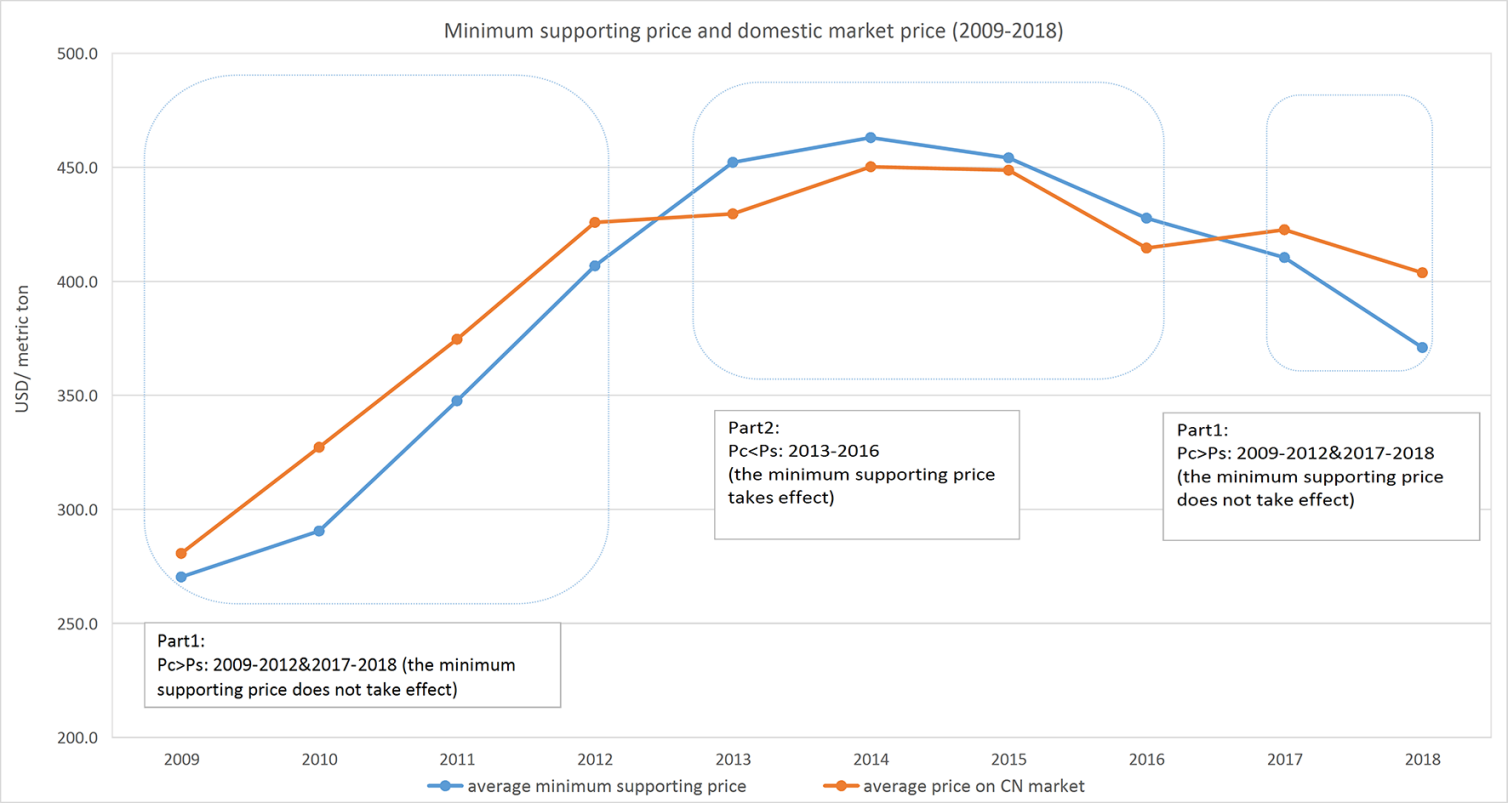
Minimum supporting price and domestic market price (2009-2018).

The most important policy in China’s price intervention program is the minimum supporting price. Since 2004, the minimum supporting price has been implemented for rice to maintain national food security and increase farmers’ incomes.^3^ Because of the increased total supply of rice, the Chinese government has to continuously buy rice from farmers to prevent the price from falling, even when massive stores of it already exist (Huang and Yang, 2017). **Figure 1** compares the minimum supporting price and domestic market price between 2009 and 2018.

Apart from the price intervention program, the Chinese government also implements a direct subsidy program for rice (and other grains). However, because the impact of agricultural subsidies on grain production has been shown to be negligible (Huang et al., 2011), we do not include this direct subsidy in the ESM.

We divide the 10-year period in **Figure 1** into two parts. Part 1 consists of the periods when the minimum price was lower than the domestic price (2009 to 2012 and 2017 to 2018), in which case the minimum price did not take effect. Part 2 consists of the period when the minimum price exceeded the domestic price (2013 to 2016).

We assume that the rest of the world (ROW) agrees to trade in Bt rice but that it does not locally cultivate it.^4^ The technology spill-over arises when the ROW follows China’s adoption of Bt rice by also locally cultivating it. When the ROW cultivates Bt rice, the ROW supply curve shifts to the right, although typically not as much as the domestic Chinese supply does (Alston et al., 1998). The technology spill-over has an effect in China and the ROW by decreasing the world price. A lower world price benefits consumers in both China and the ROW, but producers in China lose due to the spill-over.

For the ESM to include the external costs of pesticide use that were introduced above, we assume there are no further research costs after 2009, since that was when the biosafety certificates of Bt rice were issued. Based on this assumption, the potential annual net benefits are the sum of foregone economic^5^ and environmental benefits. This means that the potential annual net benefits (*AB_t_*) after commercialization are equal to the sum of the change of annual welfare (∆*TS_t_*) and annual external costs of pesticides (*TEC_pt_*):

$$AB_{t}=∆TS_{t}+{\mathrm{TEC}}_{\mathrm{pt}},$$

where *t* denotes the year (*t* = 0 corresponds to 2009).

We calculate the net present value of the potential annual benefits in 2009 and 2019 using the following equations:

$${\mathrm{NPV}}_{2009}=\sum_{t=0}^{\infty }{\left(1+\mu \right)}^{-t}AB_{t}$$

and

$${\mathrm{NPV}}_{2019}=\sum_{t=10}^{\infty }{\left(1+\mu \right)}^{-t}AB_{t},$$

where μ denotes the discount rate of an infinite stream of annual benefits. The CoP is then given by the difference between *NPV*_2009_ and *NPV*_2019_.

## Data Sources

The data come from both primary and secondary sources. The primary data are from the preproduction trial of Bt rice in China (R. Hu, private communication, 2017) and include the maximum adoption rate, yield, and input costs (**Appendix 1**). For the ESM, we calculate proportionate yield change and proportionate input cost change (per hectare) based on pesticide cost, labor cost, seed cost, fertilizer cost, and other costs. Because it takes time for farmers to adopt a new technology, we employ a logistic adoption function with a 55% ceiling.

All the secondary data come from official statistics and the literature (**Table 1**). The rice supply elasticity and the rice demand elasticity for China are based on Zhuang and Abbott (2007). The rice supply elasticity and rice demand elasticity for the ROW are based on Mohanty et al. (2017). The domestic price is from ChinaGrain (2018), and the minimum supporting price is from the Ministry of Agriculture and Rural Affairs (MARA). Because we do not have data on rice stocks, we assume that, in both China and the ROW, the annual consumption and production of rice are equal after adjusting for trade. The data on domestic production are available for the period from 2009 to 2016 from the official website of the NBSC. The rice production quantity for the ROW is available for the period from 2009 to 2016 from the Rice Yearbook of the USDA. For the remaining 3 years for which data are not yet available, we assume the quantities are the same as in 2016 (the same holds for prices after 2018).

**Table 1:** Parameterization and simulated results for the Economic Surplus Model in the period 2009-2019.

Raw parameters	unit	2009	2010	2011	2012	2013	2014	2015	2016	2017	2018	2019*	Source
Initial domestic rice price	US dollar/ton	280.69	327.25	374.63	425.86	429.61	450.25	448.76	414.61	422.65	403.77	403.77	ChinaGrain (2018)
Initial CN rice consumption	million tons	194.67	195.53	201.08	206.33	205.41	205.41	211.32	210.15	210.15	210.15	210.15	NBSC (2018)
Initial CN rice production	million tons	195.1	195.76	201	204.24	203.61	203.61	208.23	207.08	207.08	207.08	207.08	NBSC (2018)
Initial ROW rice consumption	million tons	656.2	672.71	697.8	695.71	712.68	711.12	700.43	713.3	713.3	713.3	713.3	USDA (2018)
Initial ROW rice production	million tons	655.77	672.47	697.88	697.8	710.89	712.91	703.52	716.38	716.38	716.38	716.38	USDA (2018)
Supply shift relative to the initial equilibrium	unit free	0.02	0.11	0.2	0.2	0.19	0.18	0.17	0.16	0.15	0.14	0.13	R. Hu, private comm. (2017)
**Simulated results**													
Equilibrium rice price after adopting new technology	US dollar/ton	280.10	323.17	366.15	416.11	419.80	440.29	438.95	406.40	415.46	397.36	397.82	
CN rice consumption after adopting new technology	million tons	194.82	196.39	202.68	207.99	207.05	207.00	212.94	211.62	211.41	211.33	211.24	
CN rice production after adopting new technology	million tons	195.98	201.03	210.92	214.30	213.82	213.51	217.94	216.00	214.74	214.23	213.72	
ROW rice consumption after adopting new technology	million tons	656.60	675.14	702.39	700.34	715.61	715.70	704.89	713.03	716.83	716.60	716.36	
ROW rice production after adopting new technolog	million tons	655.44	670.50	694.15	694.03	708.84	709.19	699.89	713.03	713.50	713.69	713.88	

2019* Means that we use the value of the latest year to estimate the value in 2019.

Based on the data above, we calibrate the intercepts and slopes of supply curves and demand curves in China and the ROW. We use the calibrated parameters to simulate the new equilibrium price after commercializing Bt rice as well as new equilibrium quantities for the production and consumption in China and the ROW.

Since the biosafety certificates of Bt rice were already issued in 2009, we set the probability of success to 1, meaning that the new technology has already been successful in reality. For the same reason, we assume there are no further research costs after 2009. As for the discount rate, in our analysis, we apply both 3% and 5% rates to see the implications for the stream of benefits and costs from 2009 to 2019 (Bayer et al., 2010).

Tabashnik (2015) notes that some of the environmental, health, and economic benefits of Bt crops fade over time due to the evolution of pest resistance. We take this effect into account by considering a technology depreciation factor. For lack of data, we adopt the depreciation factor for Bt eggplant (Bayer, 2007). The factor equals one in the first 4 years. Starting in the fifth year, it decreases by five percentage points annually until it reaches 65%; from then, it remains constant at that level.

To calculate the external costs of pesticide use, we choose the three most commonly used rice pesticides in China (China Agrochemical Industry Network, 2012): Imidacloprid, Cartap hydrochloride, and Chlorantraniliprole. The percentage of the active ingredient of a certain pesticide and its application rates come from the product instructions. The base value of the external cost is calculated by Leach and Mumford (2008), and we use the US Inflation Calculator^6^ to convert it to 2009 US dollars. We use the Environmental Impact Quotient (EIQ) calculator^7^ to get the EIQ values for the three pesticides. We compare these values with the reference values for each category (Leach and Mumford, 2008) and determine whether a pesticide has a low, medium, or high level of toxicity. Based on the data from the World Bank (2018) and the NBSC (2009), we compare the ratio of China’s share of employment in agriculture to the average share of agricultural employment in Germany, the United Kingdom (UK), and the US (weighted by gross domestic product [GDP]). We also compare the ratio of China’s GDP per capita to the weighted average GDP per capita in Germany, the UK, and the US. **Appendix 2** contains the details of the calculations.

## Results

### Base Model

Using the PEA tool, we estimate the annual external costs of the uses of Chlorantraniliprole, Imidacloprid, and Cartap hydrochloride in China to be 1.8 million US dollars (0.06 dollars per hectare of agricultural land). (We calculated this amount using the equation and data presented in **Appendix 2**.) Considering that China banned a series of pesticides with a high level of toxicity in 2002,^8^ the current pesticides used for rice are relatively environmentally friendly, which is also reflected in the annual external costs of pesticides.

Considering China as a large, open economy, the CoP of commercializing Bt rice from 2009 to 2019 is 104 billion US dollars under the 3% discount rate and 94 billion US dollars under 5% discount rate. We use the capital recovery factor (CRF) to calculate the annual CoP, which considers the time value of money and converts the CoP into a stream of equal payments from 2009 to 2019 at both the 3% and 5% discount rates. Under both discount rates, China loses approximately the same amount (12 billion US dollars) annually from 2009 to 2019 (**Table 2**).

**Table 2:** Results of base model simulation (billion US dollars).

Discount rate (r)	NPV2009	NPV2019	CoP	CRF (unit free)	Annual CoP
3%	372	360	104	0.117	12.22
5%	224	212	94	0.130	12.15

Capital Recovery Factor (CRF) = [i(1+i)^n^]/[(1+i)^n^-1], where n = 2019-2009 = 10.

### Effect of the Technology Spill-Over

**Figure 2:**
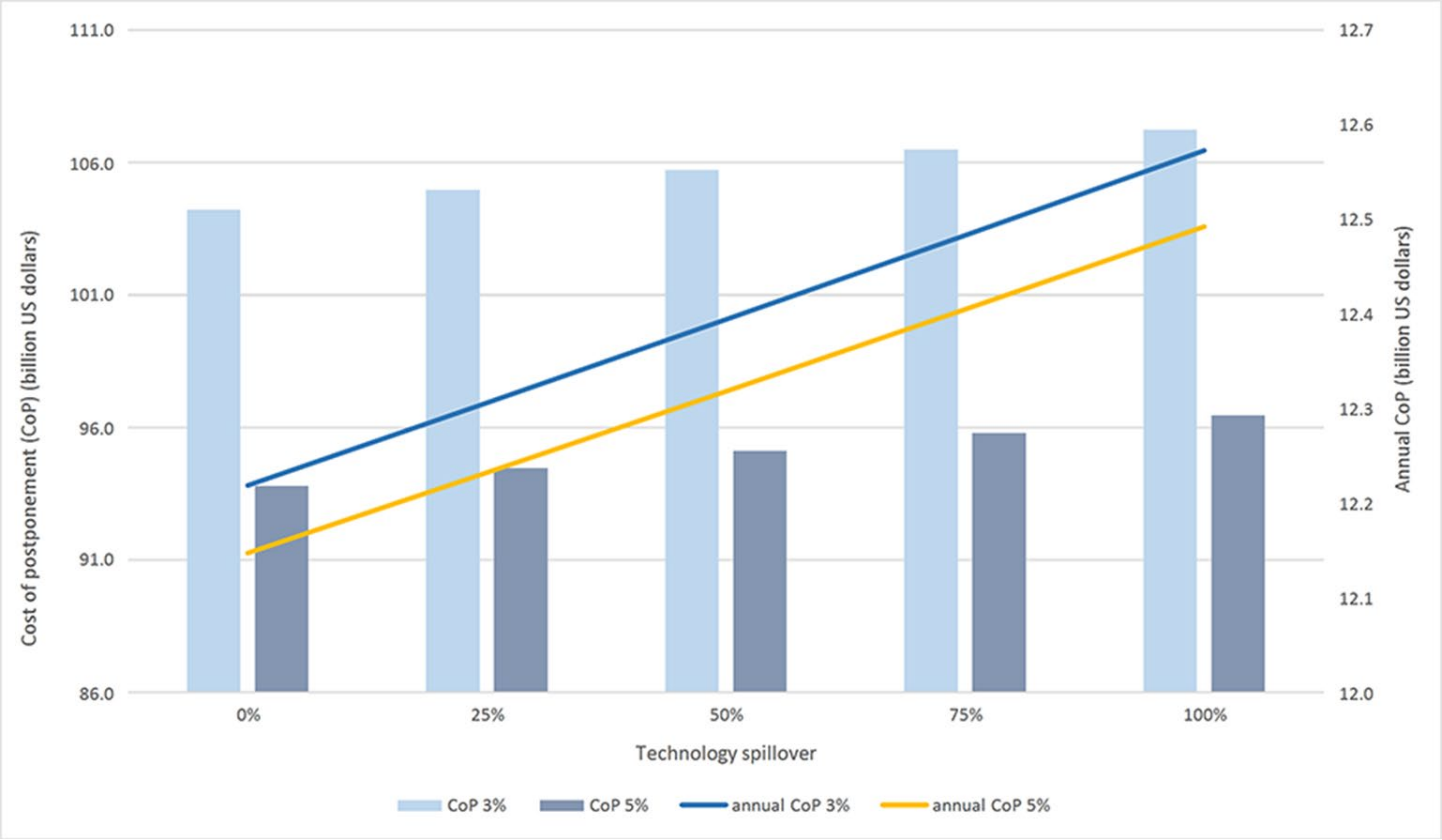
Cost of postponement and technology spillover.

Different levels of technology spill-over in the ROW have implications for economic impacts on China (**Table 3**). We assume that the ROW’s proportionate reduction in price due to the spill-over changes by 25%, 50%, 75%, and 100% compared to the base proportionate reduction in price. **Figure 2** shows the results.

**Table 3:** Sensitivity analysis of technology spillover.

	unit	2009	2010	2011	2012	2013	2014	2015	2016	2017	2018	2019*
**0% spillover**												
Rice price	US dollar/ton	280.10	323.17	366.15	416.11	419.80	440.29	438.95	406.40	415.46	397.36	397.82
CN rice consumption	million tons	194.82	196.39	202.68	207.99	207.05	207.00	212.94	211.62	211.41	211.33	211.24
CN rice production	million tons	195.98	201.03	210.92	214.30	213.82	213.51	217.94	216.00	214.74	214.23	213.72
ROW rice consumption	million tons	656.60	675.14	702.39	700.34	715.61	715.70	704.89	717.41	716.83	716.60	716.36
ROW rice production	million tons	655.44	670.50	694.15	694.03	708.84	709.19	699.89	713.03	713.50	713.69	713.88
**25% spillover**												
Rice price	US dollar/ton	279.95	322.17	364.08	413.73	417.41	437.86	436.55	404.39	413.69	395.78	396.35
CN rice consumption	million tons	194.85	196.60	203.08	208.39	207.46	207.40	213.34	211.97	211.72	211.61	211.51
CN rice production	million tons	195.95	200.86	210.62	213.99	213.82	213.51	217.94	216.00	214.50	214.01	213.51
ROW rice consumption	million tons	656.70	675.74	703.51	701.48	716.76	716.81	705.98	718.42	717.70	717.41	717.11
ROW rice production	million tons	655.36	670.01	693.24	693.11	707.90	708.28	699.00	712.21	712.79	713.03	713.27
**50% spillover**												
Rice price	US dollar/ton	279.81	321.16	362.01	411.34	415.01	435.42	434.15	402.38	411.92	394.20	394.88
CN rice consumption	million tons	194.89	196.81	203.47	208.80	207.86	207.79	213.74	212.33	212.03	211.90	211.78
CN rice production	million tons	195.92	200.70	210.32	213.68	213.82	213.51	217.94	216.00	214.27	213.79	213.31
ROW rice consumption	million tons	656.80	676.34	704.64	702.61	717.91	717.93	707.07	719.42	718.57	718.22	717.87
ROW rice production	million tons	655.28	669.52	692.33	692.18	706.97	707.37	698.11	711.39	712.09	712.37	712.66
**75% spillover**												
Rice price	US dollar/ton	279.66	320.16	359.94	408.96	412.62	432.99	431.75	400.37	410.16	392.63	393.42
CN rice consumption	million tons	194.93	197.02	203.86	209.21	208.26	208.18	214.13	212.69	212.34	212.19	212.05
CN rice production	million tons	195.89	200.53	210.01	213.37	213.82	213.51	217.94	216.00	214.03	213.57	213.10
ROW rice consumption	million tons	656.90	676.94	705.76	703.74	719.06	719.05	708.16	720.43	719.43	719.03	718.62
ROW rice production	million tons	655.20	669.04	691.42	691.26	706.03	706.46	697.22	710.57	711.38	711.71	712.04
**100% spillover**												
Rice price	US dollar/ton	279.51	319.15	357.87	406.58	410.22	430.55	429.35	398.36	408.39	391.05	391.95
CN rice consumption	million tons	194.96	197.23	204.25	209.61	208.67	208.57	214.53	213.05	212.65	212.48	212.32
CN rice production	million tons	195.86	200.37	209.71	213.06	213.82	213.51	217.94	216.00	213.79	213.35	212.90
ROW rice consumption	million tons	657.00	677.55	706.88	704.87	720.22	720.17	709.25	721.44	720.30	719.84	719.38
ROW rice production	million tons	655.11	668.55	690.51	690.34	705.09	705.55	696.34	709.75	710.67	711.05	711.43

2019* means that we use the value of the latest year to estimate the value in 2019.

With the increase in technology spill-over, the world rice price decreases. The lower world price benefits consumers in both China and the ROW. During the 10 years under study, China was a net importer in all years except for 2009 and 2010. **Figure 2** shows the effects of technology spill-over during this 10-year period. The total and annual CoP both increase when the level of technology spill-over increases. The percentage change in CoP is small, however. For example, at both 3% and 5% discount rates, the annual CoP increases by around 350 million US dollars when the technology spill-over rises from 0% to 100%: The relative change from the initial value is less than 3%.

### Effects of the Maximum Adoption Rate and the Rate of Diffusion

We model the annual adoption rate (*A_t_*) for Bt rice using the logistic function

$$\mathrm{At}=\frac{\rho_{\text{max}}}{1+e^{-a-\beta t}},$$

where ρ_max_ denotes the maximum adoption rate, α represents a constant of integration, and the parameter β represents the rate of diffusion, which measures the rate at which adoption *A_t_* increases with time *t* (Alston et al., 1998).

**Figure 3:**
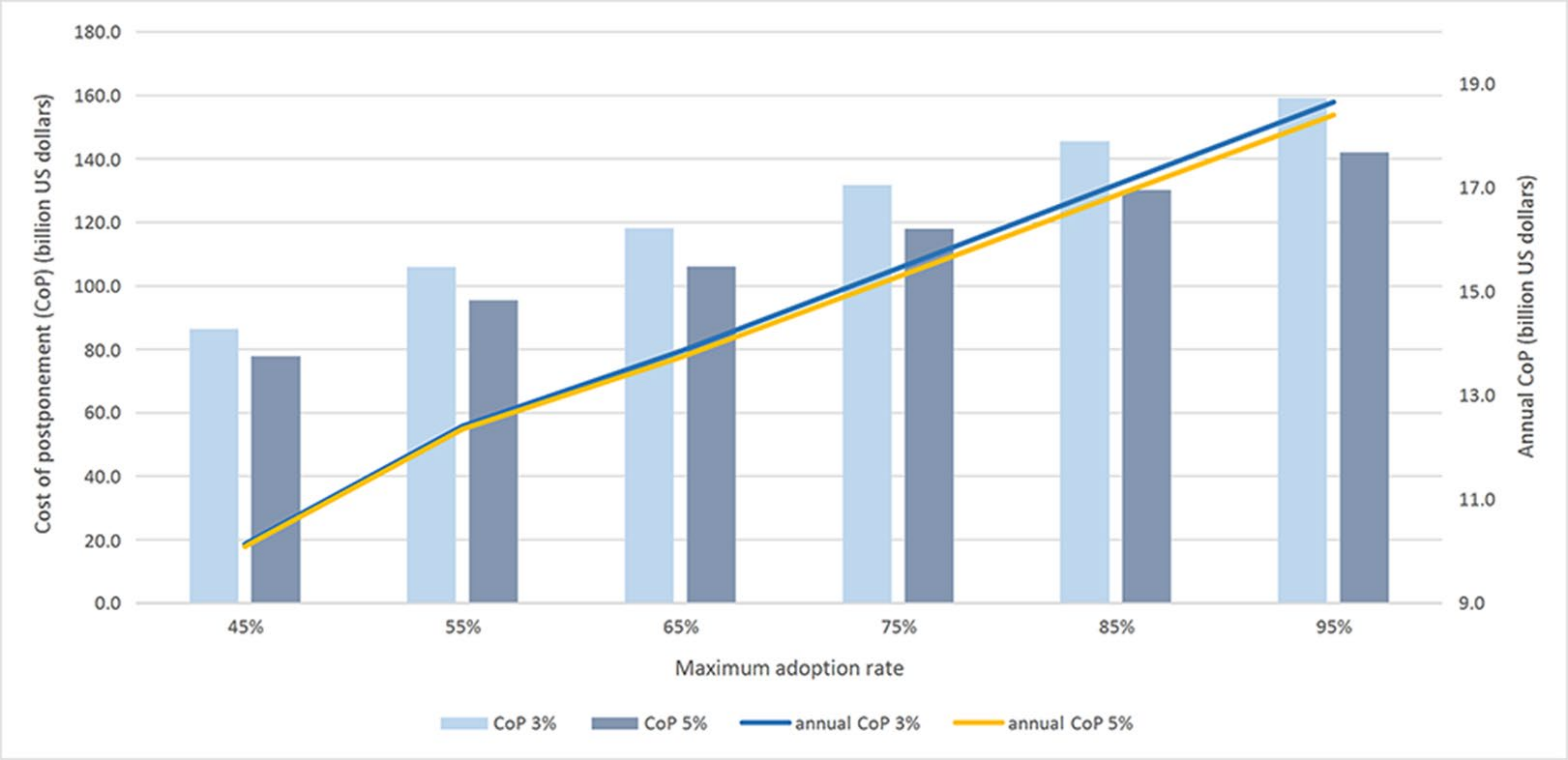
Cost of postponement and maximum adoption rate.

For the maximum adoption rate, no data are available, since Bt rice has not been approved for cultivation yet. The maximum adoption rate we use in the baseline is 55%, which corresponds to a preproduction trial (R. Hu, private communication, 2017). We assume the adoption rate for the first year is 5% (*A_1_* = 0.05). Since it took 3 years for the adoption rate to reach 55% in the preproduction trial in the period from 2002 to 2004, we set *A_3_* = 0.54 under the assumption that the adoption rate almost reached its maximum. Based on these assumptions, the calibrated parameters are α = −5.45 and β = 3.15. In further sensitivity analyses (**Figure 3** and **Table 4**), we set the maximum adoption rate to 0.45, 0.55, 0.65, 0.75, 0.85, and 0.95 (and recalibrate the parameters α and β accordingly).

**Table 4:** Sensitivity analysis of maximum adoption rate.

	unit	2009	2010	2011	2012	2013	2014	2015	2016	2017	2018	2019*
**45% maximum adoption rate**												
α	unit free	-5.01	-5.01	-5.01	-5.01	-5.01	-5.01	-5.01	-5.01	-5.01	-5.01	-5.01
β	unit free	2.93	2.93	2.93	2.93	2.93	2.93	2.93	2.93	2.93	2.93	2.93
Adoption rate	unit free	0.05	0.32	0.44	0.45	0.45	0.45	0.45	0.45	0.45	0.45	0.45
Rice price	US dollar/ton	280.10	322.96	367.85	417.89	421.59	442.10	440.73	407.89	416.76	398.52	398.90
CN rice consumption	million tons	194.82	196.43	202.36	207.68	206.75	206.71	212.65	211.35	211.18	211.11	211.04
CN rice production	million tons	195.98	201.30	208.94	212.46	211.96	211.71	216.17	214.37	213.35	212.93	212.51
ROW rice consumption	million tons	656.60	675.27	701.47	699.50	714.75	714.86	704.07	716.66	716.19	716.00	715.80
ROW rice production	million tons	655.44	670.40	694.90	694.72	709.54	709.87	700.55	713.64	714.02	714.18	714.34
**55% maximum adoption rate**												
α	unit free	-5.45	-5.45	-5.45	-5.45	-5.45	-5.45	-5.45	-5.45	-5.45	-5.45	-5.45
β	unit free	3.15	3.15	3.15	3.15	3.15	3.15	3.15	3.15	3.15	3.15	3.15
Adoption rate	unit free	0.05	0.38	0.54	0.55	0.55	0.55	0.55	0.55	0.55	0.55	0.55
Rice price	US dollar/ton	280.10	322.03	366.30	416.12	419.80	440.29	438.95	406.40	415.46	397.36	397.82
CN rice consumption	million tons	194.82	196.63	202.66	207.99	207.05	207.00	212.94	211.62	211.41	211.33	211.24
CN rice production	million tons	195.98	202.51	210.74	214.30	213.82	213.51	217.94	216.00	214.74	214.23	213.72
ROW rice consumption	million tons	656.60	675.83	702.31	700.34	715.61	715.70	704.89	717.41	716.83	716.60	716.36
ROW rice production	million tons	655.44	669.94	694.22	694.03	708.84	709.19	699.89	713.03	713.50	713.69	713.88
**65% maximum adoption rate**												
α	unit free	-4.58	-4.58	-4.58	-4.58	-4.58	-4.58	-4.58	-4.58	-4.58	-4.58	-4.58
β	unit free	2.09	2.09	2.09	2.09	2.09	2.09	2.09	2.09	2.09	2.09	2.09
Adoption rate	unit free	0.05	0.26	0.55	0.64	0.65	0.65	0.65	0.65	0.65	0.65	0.65
Rice price	US dollar/ton	280.10	323.68	366.15	414.59	418.05	438.48	437.17	404.91	414.15	396.19	396.73
CN rice consumption	million tons	194.82	196.28	202.68	208.25	207.35	207.29	213.24	211.88	211.64	211.54	211.44
CN rice production	million tons	195.98	200.37	210.92	215.87	215.64	215.31	219.71	217.62	216.13	215.53	214.93
ROW rice consumption	million tons	656.60	674.84	702.39	701.07	716.45	716.53	705.70	718.16	717.47	717.19	716.92
ROW rice production	million tons	655.44	670.75	694.15	693.44	708.15	708.51	699.23	712.42	712.98	713.20	713.43
**75% maximum adoption rate**												
α	unit free	-4.46	-4.46	-4.46	-4.46	-4.46	-4.46	-4.46	-4.46	-4.46	-4.46	-4.46
β	unit free	1.83	1.83	1.83	1.83	1.83	1.83	1.83	1.83	1.83	1.83	1.83
Adoption rate	unit free	0.05	0.23	0.55	0.71	0.74	0.75	0.75	0.75	0.75	0.75	0.75
Rice price	US dollar/ton	280.10	324.12	366.15	413.30	416.36	436.69	435.38	403.42	412.84	395.03	395.65
CN rice consumption	million tons	194.82	196.19	202.68	208.47	207.63	207.58	213.53	212.15	211.87	211.75	211.64
CN rice production	million tons	195.98	199.80	210.92	217.21	217.40	217.09	221.47	219.24	217.53	216.83	216.13
ROW rice consumption	million tons	656.60	674.57	702.39	701.68	717.26	717.35	706.50	718.90	718.11	717.79	717.47
ROW rice production	million tons	655.44	670.96	694.15	692.94	707.49	707.84	698.57	711.81	712.45	712.72	712.98
**85% maximum adoption rate**												
α	unit free	-4.46	-4.46	-4.46	-4.46	-4.46	-4.46	-4.46	-4.46	-4.46	-4.46	-4.46
β	unit free	1.69	1.69	1.69	1.69	1.69	1.69	1.69	1.69	1.69	1.69	1.69
Adoption rate	unit free	0.05	0.21	0.55	0.77	0.83	0.85	0.85	0.85	0.85	0.85	0.85
Rice price	US dollar/ton	280.10	324.33	366.15	412.17	414.73	434.91	433.61	401.93	411.54	393.86	394.57
CN rice consumption	million tons	194.82	196.14	202.68	208.66	207.91	207.87	213.83	212.41	212.10	211.97	211.84
CN rice production	million tons	195.98	199.53	210.92	218.37	219.10	218.86	223.23	220.86	218.92	218.13	217.34
ROW rice consumption	million tons	656.60	674.45	702.39	702.22	718.05	718.17	707.31	719.65	718.76	718.39	718.03
ROW rice production	million tons	655.44	671.06	694.15	692.50	706.86	707.18	697.91	711.20	711.93	712.23	712.52
**95% maximum adoption rate**												
α	unit free	-4.49	-4.49	-4.49	-4.49	-4.49	-4.49	-4.49	-4.49	-4.49	-4.49	-4.49
β	unit free	1.60	1.60	1.60	1.60	1.60	1.60	1.60	1.60	1.60	1.60	1.60
Adoption rate	unit free	0.05	0.21	0.55	0.83	0.92	0.94	0.95	0.95	0.95	0.95	0.95
Rice price	US dollar/ton	280.10	324.45	366.15	411.16	413.16	433.15	431.83	400.44	410.23	392.70	393.49
CN rice consumption	million tons	194.82	196.12	202.68	208.83	208.17	208.15	214.12	212.68	212.32	212.18	212.03
CN rice production	million tons	195.98	199.37	210.92	219.41	220.74	220.61	224.98	222.48	220.31	219.43	218.55
ROW rice consumption	million tons	656.60	674.38	702.39	702.69	718.81	718.98	708.12	720.40	719.40	718.99	718.59
ROW rice production	million tons	655.44	671.12	694.15	692.12	706.24	706.52	697.25	710.60	711.41	711.74	712.07

2019* means that we use the value of the latest year to estimate the value in 2019.

**Figure 4:**
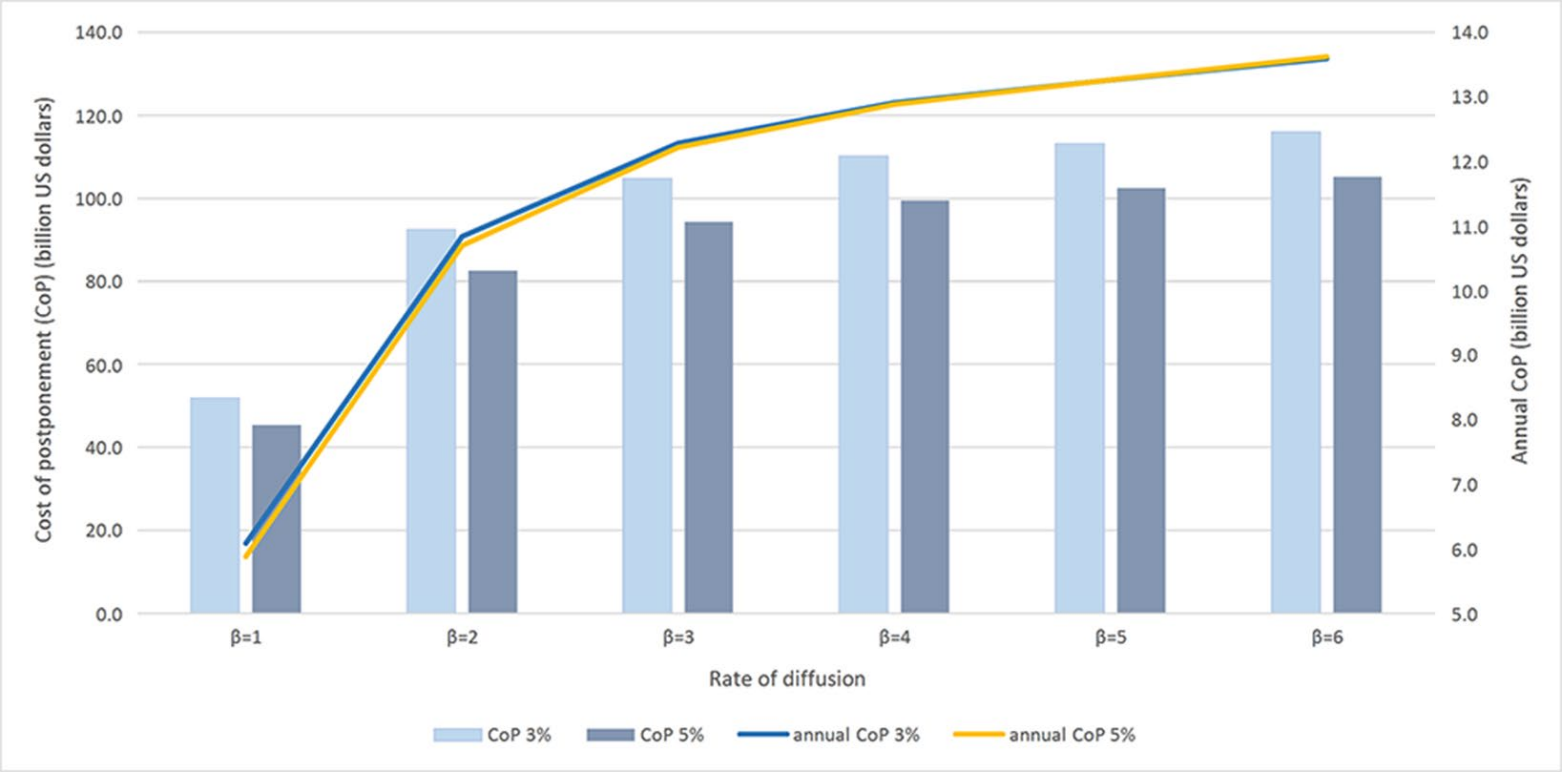
Cost of postponement and rate of diffusion.

In another set of sensitivity analyses (**Figure 4** and **Table 5**), we examine the effect of the rate of diffusion (ʑβ) on CoP (holding ρ*^max^* and α at their baseline levels) because the speed of adopting new technology is important when the cultivation area is large. We vary the parameter β between 1 and 6.

**Table 5:** Sensitivity analysis of rate of diffusion.

	unit	2009	2010	2011	2012	2013	2014	2015	2016	2017	2018	2019*
**β=1**												
Rice price	US dollar/ton	397.84	327.02	373.95	424.00	425.79	443.93	440.66	407.00	415.66	397.43	397.84
CN rice consumption	million tons	211.24	195.58	201.21	206.64	206.05	206.42	212.66	211.51	211.37	211.31	211.24
CN rice production	million tons	213.69	196.06	201.79	206.15	207.59	209.89	216.24	215.35	214.53	214.15	213.69
ROW rice consumption	million tons	716.35	672.84	698.16	696.59	712.72	714.02	704.11	717.11	716.73	716.56	716.35
ROW rice production	million tons	713.89	672.36	697.58	697.08	711.18	710.55	700.52	713.27	713.58	713.72	713.89
**β=2**												
Rice price	US dollar/ton	280.49	325.83	369.25	416.81	419.91	440.31	438.95	406.40	415.46	397.36	397.82
CN rice consumption	million tons	194.72	195.83	202.10	207.87	207.04	207.00	212.94	211.62	211.41	211.33	211.24
CN rice production	million tons	195.40	197.60	207.30	213.58	213.71	213.50	217.94	215.99	214.74	214.23	213.72
ROW rice consumption	million tons	656.33	673.55	700.71	700.01	715.56	715.69	704.88	717.41	716.83	716.60	716.36
ROW rice production	million tons	655.66	671.78	695.51	694.30	708.88	709.19	699.89	713.03	713.50	713.69	713.88
**β=3**												
Rice price	US dollar/ton	280.18	322.51	366.39	416.12	419.80	440.29	438.95	406.40	415.46	397.36	397.82
CN rice consumption	million tons	194.80	196.53	202.64	207.99	207.05	207.00	212.94	211.62	211.41	211.33	211.24
CN rice production	million tons	195.87	201.89	210.65	214.29	213.82	213.51	217.94	216.00	214.74	214.23	213.72
ROW rice consumption	million tons	656.55	675.54	702.26	700.34	715.60	715.70	704.89	717.41	716.83	716.60	716.36
ROW rice production	million tons	655.48	670.18	694.26	694.03	708.84	709.19	699.89	713.03	713.50	713.69	713.88
**β=4**												
Rice price	US dollar/ton	279.46	320.32	366.16	416.11	419.80	440.29	438.95	406.40	415.46	397.36	397.82
CN rice consumption	million tons	194.98	196.99	202.68	207.99	207.05	207.00	212.94	211.62	211.41	211.33	211.24
CN rice production	million tons	196.93	204.72	210.91	214.30	213.82	213.51	217.94	216.00	214.74	214.23	213.72
ROW rice consumption	million tons	657.04	676.85	702.39	700.34	715.61	715.70	704.89	717.41	716.83	716.60	716.36
ROW rice production	million tons	655.08	669.11	694.16	694.03	708.84	709.19	699.89	713.03	713.50	713.69	713.88
**β=5**												
Rice price	US dollar/ton	278.16	319.86	366.15	416.11	419.80	440.29	438.95	406.40	415.46	397.36	397.82
CN rice consumption	million tons	195.29	197.08	202.68	207.99	207.05	207.00	212.94	211.62	211.41	211.33	211.24
CN rice production	million tons	198.85	205.32	210.92	214.30	213.82	213.51	217.94	216.00	214.74	214.23	213.72
ROW rice consumption	million tons	657.92	677.12	702.39	700.34	715.61	715.70	704.89	717.41	716.83	716.60	716.36
ROW rice production	million tons	654.37	668.89	694.15	694.03	708.84	709.19	699.89	713.03	713.50	713.69	713.88
**β=6**												
Rice price	US dollar/ton	276.57	319.79	366.15	416.11	419.80	440.29	438.95	406.40	415.46	397.36	397.82
CN rice consumption	million tons	195.68	197.10	202.68	207.99	207.05	207.00	212.94	211.62	211.41	211.33	211.24
CN rice production	million tons	201.20	205.40	210.92	214.30	213.82	213.51	217.94	216.00	214.74	214.23	213.72
ROW rice consumption	million tons	659.00	677.16	702.39	700.34	715.61	715.70	704.89	717.41	716.83	716.60	716.36
ROW rice production	million tons	653.49	668.86	694.15	694.03	708.84	709.19	699.89	713.03	713.50	713.69	713.88

2019* Means that we use the value of the latest year to estimate the value in 2019.

Both figures confirm that the economic benefits are larger the more farmers adopt Bt rice and the faster they adopt it. For example, when the maximum adoption rate increases by 10% (from 55% to 65%), the annual CoP increases by around 1.5 billion dollars. When the rate of diffusion gets larger, the speed of the increase in both CoP and annual CoP gets smaller. At both 3% and 5% discount rates, the annual C_O_P doubles when the rate of diffusion changes from 1 to 6.

### Actionable Recommendations

The results show that the continuous postponement of Bt rice introduction in China has come at a substantial economic cost that includes not only the direct economic losses of efficiency at higher prices of rice for consumers but also human health and environmental costs.

These costs have to be weighed against consumer concerns about Bt rice. Consumers, including those in China, tend to ignore the environmental benefits of crop production in their purchasing behavior. The introduction of Bt rice in combination with information about its environmental benefits, such as lower pesticide use and reduced greenhouse gas emission (Wesseler et al., 2011), may overcome some of the potential consumer resistance. Further, linking the introduction of Bt rice with a labelling policy might also increase consumer acceptance, as reported, for example, in the US (Kolodinsky and Lusk, 2018).

Our study suggests two main actionable policy recommendations. First, as further delays in the approval for Bt rice cultivation results in substantial costs, it should immediately be approved for cultivation. Second, for addressing potential consumer concerns, its introduction should be accompanied by a mandatory labelling of consumer products derived from Bt rice.

An additional policy recommendation is to link the approval of Bt rice cultivation with an information campaign about its environmental benefits. Further, Bt rice is just one example among several new crops developed using advances in plant breeding. The results presented for Bt rice carry over to many other crops, including Vitamin A-enriched rice (Wesseler and Zilberman, 2014), insect-resistant vegetables, such as eggplants and tomatoes (Groeneveld et al., 2011), and GMOs in general (Barrows et al., 2014). Studies show that delaying approval for the cultivation of these crops comes at substantial economic costs (see, for example, Zilberman et al., 2018). They not only directly benefit both farmers and consumers but also substantially benefit the environment, including, in some cases, substantial reductions in greenhouse gas emissions (Smyth et al., 2011). Policymakers in China should take these implications more explicitly into consideration when determining the approval of Bt rice and other crops developed using advanced plant-breeding technologies.

## Discussion

So far, no study has reported any adverse side effects of consuming food products derived from GM crops anywhere in the world (Paarlberg, 2009). Many scientific studies, to the contrary, present evidence that GM crops can be safely used in food and feed and are nutritionally equivalent to their non-GM counterparts (Snell et al., 2012; Bawa and Anilakumar, 2013). This also holds for the case of Bt rice (Li et al., 2014; Li et al., 2016).

As a major producer, consumer, and trader of rice, China issued biosafety certificates for Bt rice in October 2009, which were renewed in December 2014, until the end of 2019; however, the commercialization of Bt rice in China has been continuously postponed and is still pending. We estimate the forgone benefits due to this postponement to be around 12 billion US dollars per year in the studied period (2009 to 2019).

This postponement is largely due to the low level of understanding and acceptance of GM crops in China (Li et al., 2016). Other challenges in commercializing Bt rice include resolving trade policy impediments and developing insect resistance management strategies (High et al., 2004; Liu et al., 2016). In January 2018, the US Food and Drug Administration and the US Environmental Protection Agency declared that Bt rice was not more dangerous than conventional rice and received legal clearance for import and consumption in the United States, indicating that Bt rice is likely to be approved in other countries in the future.

An important limitation of Bt rice is that it was developed to control lepidopteran pests but no other rice pests. Also, some lepidopteran pests are likely to increase their resistance to Bt rice after commercialization (Li et al., 2014); therefore, insect resistance management strategies are required before commercializing Bt rice. However, waiting for the identification of new genes to control non-lepidopteran pests or the development of new plant breeding technologies might result in sunk research and investment costs in Bt rice.

## Data Availability Statement

All datasets generated for this study are included in the manuscript/**Supplementary Files**.

## Author Contributions

YJ performed the calculations. DD and NH verified the analytical methods. JW helped shape the research and analysis.

## Funding

Yan Jin has received financial support for this project from the China Scholarship Council. The Agricultural Economics and Rural Policy Group of Wageningen University has kindly paid for the article processing fee.

## Conflict of Interest

The authors declare that the research was conducted in the absence of any commercial or financial relationships that could be construed as a potential conflict of interest.

## Footnotes

1 The Ministry of Agriculture (MoA) transformed into the Ministry of Agriculture and Rural Affairs (MARA) in early 2018. The main difference after the transformation is the integration of sections of agricultural investment and management from different ministries, such as the Ministry of Finance and the Ministry of Land and Resources, into MARA. More details (in Chinese) are available at http://www.npc.gov.cn/npc/xinwen/2018-03/18/content_2050371.htm. The role of MARA in regulating genetically modified organisms (GMOs) has not changed significantly in comparison to the role of MoA.

2 http://www.gov.cn/zhengce/content/2016-08/08/content_5098072.htm (in Chinese).

3 For wheat in 2006 and maize in 2008.

4 Recent developments support this assumption. For example, in January 2018, Bt rice was approved by the US Food and Drug Administration (FDA, 2018) and Environmental Protection Agency (EPA). This approval means that Bt rice can be consumed and imported to the United States but cannot be cultivated there.

5 In the model, we use the annual total production of rice in the rice seasons (single or double-cropping rice) that have been taken into consideration. Therefore, different rice seasons in different regions in China will not influence the results. An important limitation of Bt rice is that it is developed to control lepidopteran pests but no other rice pests, such as plant hoppers. Herbicide is still needed for Bt rice to control weeds. Field trials of Bt rice revealed that pesticide is still needed (for non-lepidopteran pests) but that its amount could decrease significantly due to the resistance of Bt rice to lepidopteran pests.

6 https://www.usinflationcalculator.com/

7 https://nysipm.cornell.edu/eiq/calculator-field-use-eiq/

8 http://www.chinapesticide.org.cn/fgzcwj/906.jhtml

## Appendices

**Appendix 1:** Parametrization for the Economic Surplus Model.

Parameter	Description	Values and unit	Source
***E(Y)***	Proportionate yield change	0.045 (per hectare)	Personal communication
***E(C)***	Proportionate change in input cost	−21.4% (per hectare)	Personal communication
***r***	Discount rate (ESM)	discuss at 3% and 5%	Bayer et al. (2010)
εa	Domestic rice supply elasticity	0.273	Zhuang and Abbott (2007)
ηa	Domestic rice demand elasticity	−0.352	Zhuang and Abbott (2007)
εb	ROW rice supply elasticity	0.236	Mohanty et al. (2017)
ηb	ROW rice demand elasticity	−0.291	Mohanty et al. (2017)
1-δ	Depreciation factor of technology	65%	Bayer (2007)
***A*** ***q***	Maximum adoption rate Probability of adopting Bt rice	55% 0.5	Personal communication Assumption

The depreciation factor of technology starts in the 5th year and drops by five percentage points annually until 65%. ESM, economic surplus model; ROW, the rest of the world.

The annual total external costs of a pesticide p (TECp) can be calculated as

$${\mathrm{TEC}}_{p}={\mathrm{rate}}_{p}\frac{{\mathrm{active}}_{p}}{100}\sum_{c=1}^{3}\left[{\mathrm{EC}}_{c}F_{c}\left(F_{\mathrm{agemp}}{|}_{c=1,2,3}\right)\right]F_{\mathrm{gdppc}},$$

where *rate_p_* denotes the application rate of a pesticide *p* in kilograms of formulated product per hectare and *active_p_* denotes the percentage of active ingredient in the formulated product (Prannetvatakul et al., 2013).

The PEA uses the Environmental Impact Quotient (EIQ) calculator to adjust the base values of economic costs to differences between relative toxicities of pesticides. There are eight events within three large categories in the EIQ with 472 active pesticide compounds in total: (*i*) farm workers,^9^ (*ii*) consumers,^10^ and (*iii*) the environment.^11^ In the study, we aggregate eight events into three categories and convert EIQ values to external costs for the three categories with subscript *c* = 1, 2, or 3 representing the categories (*i*), (*ii*), or (*iii*), respectively. The PEA tool converts EIQ values for the three categories to external costs by multiplying the external cost base values with a factor *F_c_* that has three levels: 0.5 if the pesticide has a relatively low toxicity level; 1.0 if it has a medium toxicity level; and 1.5 if it has a relatively high toxicity level. Leach and Mumford (2008) define the ranges of toxicity level for each category.

*EC_c_* is the base value of external costs calculated by Leach and Mumford (2008) converted to 2009 US dollars. The parameter *F_agemp_* denotes the ratio of China’s share of employment in agriculture to the average share of agricultural employment in the Unites States (US), the United Kingdom (UK), and Germany weighted by the gross domestic product (GDP). *F_agemp_* takes into consideration that, in China, more people are engaged in agriculture than in the other three countries, thus having more direct contact with pesticides.

The parameter *F_gdppc_* denotes the ratio of China’s GDP per capita to the average GDP per capita in the US, the UK, and Germany, weighted by the GDPs of those countries. *F_gdppc_* considers that, due to lower labor costs in China, lower costs of monitoring and cleaning up lead to lower external costs (Prannetvatakul et al., 2013).

## Appendix

**Appendix 2:** The Parametrization of the Pesticide Environmental Accounting (PEA) Tool.

	rate_p_	active_p_	EC_1_	EC_2_	EC_3_	F_1_	F_2_	F_3_	F_agemp_ (c = 1,2,3)			F_gdppc_
Chlorantraniliprole	0.001	0.2	1.8	6.09	2.76	0.5	0.5	0.5	20.243	1	1	0.085
Imidacloprid	0.041	0.7	1.8	6.09	2.76	0.5	0.5	0.5	20.243	1	1	0.085
Cartap hydrochloride	0.003	0.98	1.8	6.09	2.76	0.5	0.5	0.5	20.243	1	1	0.085
Source	Personal calculation based on data from www.taobao.com	Cornell EIQ calculator: https://nysipm.cornell.edu/eiq/calculator-field-use-eiq				Leach and Mumford (2008)			Personal calculation based on data from World Bank (2018) and NBSC (2009)			

## References

[B1] Alston J. M. Norton G. W. Pardey P. G. (1998). Science and scarcity. New York, NY: CAB International.

[B2] Barrows D. Sexton S. Zilberman D. (2014). Agricultural biotechnology: the promise and prospects of genetically modified crops. J. Econ. Perspect. 28 (1), 99–120. 10.1257/jep.28.1.99

[B3] Bawa A. S. Anilakumar K. R. (2013). Genetically modified foods: safety, risks and public concerns—a review. J. Food Sci. Technol. 50 (6), 1035–1046. 10.1007/s13197-012-0899-1 24426015 PMC3791249

[B4] Bayer J. C. (2007). Biotechnologies in the Philippines: the cost of regulation. A Master's thesis. Virginia Tech University. 1–63. Available at: https://theses.lib.vt.edu/theses/available/etd-06082007-105019/unrestricted/JBayerThesis.pdf (accessed 1 March 2018).

[B5] Bayer J. C. Norton G. W. Falck-Zepeda J. B. (2010). Cost of compliance with biotechnology regulation in the Philippines: implications for developing countries. AgBioForum 13 (1), 53–62.

[B6] China Agrochemical Industry Network (2012). Introduction of rice pesticides. (in Chinese), available at: http://www.ccpia.com.cn/info.asp?classid=L010205&newsid=L206131605000566 (accessed 1 March 2018).

[B7] ChinaGrain (2018). Domestic price of rice in China. (in Chinese), available at: http://datacenter.cngrain.com/KLineEnd.aspx?ID=18&Str=PD (accessed 1 March 2018).

[B8] Chen M. Shelton A. Ye G. Y. (2011). Insect-resistant genetically modified rice in China: from research to commercialization. Annu. Rev. Entomol. 56, 81–101. 10.1146/annurev-ento-120709-144810 20868281

[B9] Chen X. Yang C. Jia H. (2014). Issues confronting GMO crops in China. J. Huazhong Agric. Univ. 33, 115–117.

[B10] Cohen M. B. Chen M. Bentur J. S. Heong K. L. Ye G. (2008). Bt rice in Asia: potential benefits, impact, and sustainability. Prog. Biol. Control 16, 223–248. 10.1007/978-1-4020-8373-0_8

[B11] Dang C. Lu Z. Wang L. Chang X. Wang F. Yao H. (2017). Does Vt rice pose risks to non-target arthropods? Results of a meta-analysis in China. Plant Biotechnol. J. 15, 1047–1053. 10.1111/pbi.12698 28111920 PMC5506656

[B12] Deng H. Hu R. Huang J. Pray C. Jin Y. Li Z. (2017). Attitudes toward GM foods, biotechnology R&D investment and lobby activities among agribusiness firms in the food, feed, chemical and seed industries in China. China Agric. Econ. Rev. 9 (3), 385–396. 10.1108/CAER-10-2016-0162

[B13] FDA (US Food and Drug Administration), 2018, available at: https://www.fda.gov/downloads/Food/IngredientsPackagingLabeling/GEPlants/Submissions/ucm592625.pdf (accessed 1 March 2018).

[B14] Groeneveld R. Ansink E. de Wiel C. Wesseler J. (2011). Benefits and costs of biologically contained GM tomatoes and eggplants in Italy and Spain. Sustainability 3, 1265–1281. 10.3390/su3081265

[B15] High S. M. Cohen M. B. Shu Q. Altosaar I. (2004). Achieving successful deployment of Bt rice. Trends Plant Sci. 9 (6), 286–292. 10.1016/S1360-1385(04)00099-8 15165560

[B16] Huang J. Qiao F. Zhang L. Rozelle S. (2000). “*Economic and Environmental Program for Southeast Asia (EEPSEA)*,” in Farm pesticide, rice production, and human health (Singapore: IDRC).

[B17] Huang J. Hu R. Rozelle S. Pray C. (2005). Insect-resistant GM rice in farmers’ fields: assessing productivity and health effects in China. Science 308, 688–690. 10.1126/science.1108972 15860626

[B18] Huang J. Wang X. Zhi H. Huang Z. Rozelle S. (2011). Subsidies and distortions in China’s agriculture: evidence from producer-level data. Aust. J. Agric. Resour. Econ. 55, 53–71. 10.1111/j.1467-8489.2010.00527.x

[B19] Huang J. Hu R. Qiao F. Yin Y. Liu H. Huang Z. (2015). Impact of insect-resistant GM rice on pesticide use and farmers’ health in China. Sci. China 58 (5), 466–471. 10.1007/s11427-014-4768-1 25576299

[B20] Huang. J. Wang Z. Dang H. (2017). Impacts of and attitudes toward GM technology in China: challenges, policy and research implications. China Agric. Econ. Rev. 9 (3), 334–339. 10.1108/CAER-07-2017-0131

[B21] Huang J. Yang G. (2017). Understanding recent challenges and new food policy in China. Glob. Food Sec. 12, 119–126. 10.1016/j.gfs.2016.10.002

[B22] Jin Y. Drabik D. Heerink N. Wesseler J. (2019). Getting an imported GM crop approved in China. Trends Biotechnol. 37 (6), 566–569. 10.1016/j.tibtech.2019.02.004 30929862

[B23] Kolodinsky J. Lusk J. (2018). Mandatory labels can improve attitudes toward genetically engineered food. Sci. Adv. 6 (4), 1–5. 10.1126/sciadv.aaq1413 PMC6021136 29963622

[B24] Krishna V. V. Qaim M. (2007). Potential impacts of Bt eggplant on economic surplus and farmers’ health in India. Agric. Econ. 38, 167–180. 10.1111/j.1574-0862.2008.00290.x

[B25] Leach A. W. Mumford J. D. (2008). Pesticide environmental accounting: a method for assessing the external costs of individual pesticide applications. Environ. Pollut. 15 (1), 139–147. 10.1016/j.envpol.2007.02.019 17604888

[B26] Leach A. W. Mumford J. D. (2011). Pesticide environmental accounting: a decision-making tool estimating external costs of pesticides. J. Consum. Prot. Food Saf. 6 (1), 521–526. 10.1007/s00003-011-0674-7

[B27] Li B. Xu Y. Han C. Han L. Hou M. Peng Y. (2014). *Chilo suppressalis* and *Sesamia inferens* display different susceptibility responses to Cry1A insecticidal proteins. Pest Manage. Sci. 71 (10), 1433–1440. 10.1002/ps.3948 25469810

[B28] Li G. Wang Y. Liu B. Zhang G. (2014). Transgenic *Bacillus thuringiensis* (Bt) rice is safer to aquatic ecosystems than its non-transgenic counterpart. PLoS One 9 (8), 1–8. 10.1371/journal.pone.0104270 PMC4126711 25105299

[B29] Li Y. Hallerman E. Liu Q. Wu K. Peng Y. (2016). The development and status of Bt rice in China. Plant Biotechnol. J. 14 (3), 839–848. 10.1111/pbi.12464 26369652 PMC11388839

[B30] Liu Q. Hallerman E. Peng Y. Li Y. (2016). Development of Bt rice and Bt maize in China and their efficacy in target pest control. Int. J. Mol. Sci. 17 (10), 1561–1576. 10.3390/ijms17101561 PMC5085622 27763554

[B31] MoA (Ministry of Agriculture of the People’s Republic of China) (2009). List of biosafety certificates for agricultural products in 2009. (in Chinese), available at: http://www.moa.gov.cn/ztzl/zjyqwgz/spxx/ (accessed 1 March 2018).

[B32] MoA (Ministry of Agriculture of the People’s Republic of China) (2016). 12th five-year plan for economic and social development. (in Chinese), available at: http://jiuban.moa.gov.cn/zwllm/zcfg/flfg/201603/t20160307_5042893.htm (accessed 1 March 2018).

[B33] Mohanty S. Chengappa P. G. Hedge M. Ladha J. K. Baruah S. Kannan E. (2017). The future rice strategy for India. Cambridge, USA: Academic Press.

[B34] NBSC (National Bureau of Statistics of China) (2009). China statistical yearbook 2009. Beijing: China Statistics Press Available at: http://data.stats.gov.cn/ (accessed 1 March 2018).

[B35] NBSC (National Bureau of Statistics of China) (2018). China statistical yearbook 2018. Beijing: China Statistics Press Available at: http://www.stats.gov.cn/tjsj/ndsj/2018/indexeh.htm.

[B36] Niu L. Mannakkara A. Qiu L. Wang X. Hua H. Lei C. (2017). Transgenic Bt rice lines producing Cry 1Ac, Cry 2Aa or Cry 1Ca have no detrimental effects on brown planthopper and pond wolf spider. Sci. Rep. 7 (1), 1–7. 10.1038/s41598-017-02207-z 28512299 PMC5434062

[B37] Paarlberg R. (2009). Starved for science. Cambridge, USA: Harvard University Press.

[B38] Prannetvatakul S. Schreinemachers P. Pananurak P. Tipraqsa P. (2013). Pesticides, external costs and policy options for Thai agriculture. Environ. Sci. Policy 27, 103–113. 10.1016/j.envsci.2012.10.019

[B39] Qu Y. Chen Y. Hou Y. Huang K. Kang D. (2011). Survey analysis of the cognition of GMO risk and safety among Chinese public. J. China Agric. Univ. 16, 1–10.

[B40] Rozelle S. Huang J. Hu R. (2005). Genetically modified rice in China: effect on farmers—in China and California. Giannini Found. Agric. Econ. 9 (1), 2–6.

[B41] Smyth S. Gusta M. Belcher K. Phillips P. W. B. Castle D. (2011). Environmental impacts from herbicide tolerant canola production in western Canada. Agric. Syst. 104, 4013–4410. 10.1016/j.agsy.2011.01.004

[B42] Snell C. Bernheim A. Bergé J. B. Kuntz M. Pascal G. Paris A. (2012). Assessment of the health impact of GM plant diets in long-term and multigenerational animal feeding trials: a literature review. Food Chem Toxicol. 50, 1134–1148. 10.1016/j.fct.2011.11.048 22155268

[B43] Tabashnik B. E. (2015). ABCs of insect resistance to Bt. PLoS Genet. 11 (11), 1–5. 10.1371/journal.pgen.1005646 PMC4652897 26583776

[B44] USDA (US Department of Agriculture) (2016). China: agricultural biotechnology annual. available at: https://www.fas.usda.gov/data/china-agricultural-biotechnology-annual-2 (accessed 1 March 2018).

[B45] USDA (US Department of Agriculture) (2018). Rice yearbook. available at: https://www.ers.usda.gov/data-products/rice-yearbook/ (accessed 1 March 2018).

[B46] Wang Y. Zhang G. Du J. Liu B. Wang M. (2010). Influence of transgenic hybrid rice expressing a fused gene derived from cry1Ab and cry1Ac on primary insect pests and rice yield. Crop Protect. 29, 128–133. 10.1016/j.cropro.2009.10.004

[B47] Wesseler J. Smart R. D. Thomson J. Zilberman D. (2017). Foregone benefits of important food crop improvements in Sub-Saharan Africa. PloS One 12 (7), 1–12. 10.1371/journal.pone.0181353 PMC5531496 28749984

[B48] Wesseler J. Zilberman D. (2014). The economic power of the golden rice opposition. Environ. Dev. Econ. 19 (6), 724–742. 10.1017/S1355770X1300065X

[B49] Wesseler J. Scatasta S. Fall E. H. (2011). “*Genetically modified food and global welfare*,” in Environmental benefits and costs of GM crops, vol. 10 in Frontiers of economics and globalization series. Eds. Carter Colin Moschini GianCarlo Sheldon Ian (Bingley, UK: Emerald Group Publishing), 173–199. 10.1108/S1574-8715(2011)0000010012

[B50] Wong A. Y. Chan A. W. (2016). Genetically modified foods in China and the United States: a primer of regulation and intellectual property protection. Food Sci. Hum. Wellness 5, 124–140. 10.1016/j.fshw.2016.03.002

[B51] World Bank (2017a). Employment in agriculture. available at: https://data.worldbank.org/indicator/sL.AGr.empL.Zs (accessed 1 March 2018).

[B52] World Bank (2017b). Arable land (hectares per person). available at: https://data.worldbank.org/indicator/AG.LND.ARBL.HA.PC?order=wbapi_data_v (accessed 1 March 2018).

[B53] World Bank (2018). Gross domestic product 2018. available at: http://databank.worldbank.org/data/download/GDP.pdf (accessed 27 August 2019).

[B54] Xie W. Ali T. Cui Q. Huang J. (2017). Economic impacts of commercializing insect-resistant GM maize in China. China Agric. Econ. Rev. 9 (3), 340–354. 10.1108/CAER-06-2017-0126

[B55] Zhuang R. Abbott P. (2007). Price elasticities of key agricultural commodities in China. China Econ. Rev. 18 (2), 155–169. 10.1016/j.chieco.2006.02.006

[B56] Zilberman D. Kaplan S. Wesseler J. (2015). The loss from underutilizing GM technologies. AgBioForum 18 (3), 312–319.

[B57] Zilberman D. Wesseler J. Schmitz A. Gordon B. (2018). Economics of Agricultural Biotechnology. Chapter 36 in Cramer G. L. Paudel K. P. Schmitz A. (eds.) The Routledge Handbook of Agricultural Economics, Routladge 670-686.

